# Achieving automated and high-precision in situ analysis of the dimensional accuracy and dynamic deformation of 3D-printed surgical templates: an in vitro study

**DOI:** 10.1186/s40729-024-00561-y

**Published:** 2024-10-15

**Authors:** Lixing He, Bowen Qin, Rongrong Zhu, Yunxian Liu, Boya Xu, Zhe Li, Liangzhi Du

**Affiliations:** 1 Key Laboratory of Shaanxi Province for Craniofacial Precision Medicine Research, College of Stomatology, Xi’an Jiaotong University, Xi’an, 710004 China; 2 Department of Digital Oral Implantology and Prothodontics, College of Stomatology, Xi’an Jiaotong University, Xi’an, 710004 China; 3 National & Local Joint Engineering Research Center of Biodiagnosis and Biotherapy, The Second Affiliated Hospital of Xi’an Jiaotong University, Xi’an, China

**Keywords:** 3D printing, Surgical template, Coordinate-measuring machine, Dimensional accuracy, Deformation

## Abstract

**Purpose:**

To demonstrate the viability of a coordinate-measuring machine (CMM) for the geometric analysis of 3D printed surgical templates.

**Methods:**

The template was designed and modified by adding 18 cylindrical landmarks for CMM test and then classified into five groups according to the slicing software and resins (opaque and transparent): Streamflow-O, Streamflow-T, Shapeware-T, Rayware-T and Polydevs-T (N = 3). Three standing times (0 w, 1 w, and 2 w) were included to observe possible deformation. All the measurements were performed automatically by the CMM through a preset program. The Euclidian distance (dxyz) was regarded as the representation of global dimension accuracy, and displacements in the x-, y-, and z-axes were also calculated.

**Results:**

The average dxyz values of Streamflow-O, Streamflow-T, Shapeware-T, Rayware-T and Polydev-T are 32.6 μm, 31.3 μm, 56.4 μm, 96.4 μm, and 55.3 μm, respectively. Deviations were mainly induced by the upward bending of the free end region (positive direction of the z-axis). Different resins did not have a significant influence on the dimensional accuracy. Moreover, deformation appeared to be negligible after 2 weeks of storage, and the z-axis displacements were only approximately 30 μm at week 1 and 10 μm at week 2.

**Conclusions:**

The deviations of the DLP-printed template are induced mainly by z-axis displacements and are determined by the processing accuracy. After 2 weeks, the dimensional stabilities of these templates are reliable, which is encouraging for clinicians. Moreover, the CMM is preliminarily demonstrated to be a feasible tool for achieving automated geometric analysis of surgical templates.

**Supplementary Information:**

The online version contains supplementary material available at 10.1186/s40729-024-00561-y.

## Background

With the development of computer-assisted implant surgery (CAIS) technology, implant insertion under the guidance of surgical templates has become a widely used method for achieving proper implant placement, which is essential for long-term stability and favorable aesthetic outcomes [1]. At present, surgical templates are mainly fabricated through 3D printing technologies using a biocompatible photosensitive resin. This is an efficient, accurate, simplified, and customized way to materialize stereolithography (STL) or computer-aided design (CAD) files into a series of dental materials, including templates, models, restorations, and prostheses [2]. However, the main drawback of 3D printing is the inferior surface characteristics caused by the layer-by-layer deposition processing [3, 4], which likely results in templates that are less accurate and easily deformed, inevitably leading to difficulties and complications in clinical application, such as inaccurate placement and intraoperative fracture [5]. Thus, surgeons need to know the dimensional accuracy and dynamic deformation of their templates before surgery.

A coordinate measuring machine (CMM) is a precision detector that can move along three mutually perpendicular rails [6]. The measurement principle is to compute the coordinates (x, y, z) for each point on the workpiece through a dedicated data processor and then to compare them with the nominal coordinates for displacement analysis [7]. Several studies have reported that the linear accuracy of the CMM is within 1 μm on all axes [8, 9]. Pan et al. preferred the CMM as the gold standard for analyzing implant placement accuracy compared with intraoral scanners, desktop optical scanners, and cone beam computed tomography (CBCT) [10]. Moreover, CMM is also frequently utilized for repeatability tests because of its ability to program movements so that the measurements can be performed repeatedly in a reproducible manner [11]. In view of the above advantages, the use of CMMs to achieve high-precision measurement of the dimensional accuracy of printed templates and in situ observation of their possible dynamic deformation is promising. To our knowledge, this is the first study reporting the application of a CMM to the geometric analysis of implant surgical templates. In this study, we systematically analyzed three factors, 3D printers, photosensitive resins and standing time after fabrication, to demonstrate the workflow for and performance of a CMM used to measure 3D-printed implant surgical templates.

## Results

### Dimensional accuracy

**Fig. 1:**
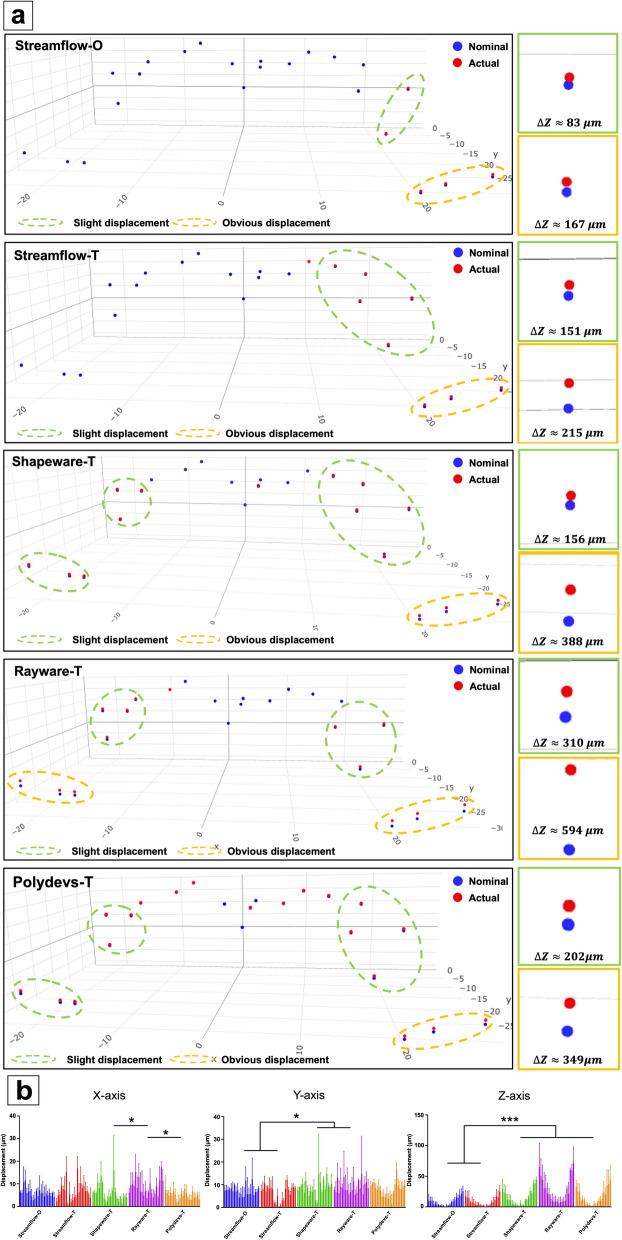
3D coordinate diagrams of all test points and their displacement comparisons along the x-, y-, and z-axes. **a** The actual (red) and nominal (blue) coordinates of all test points are simultaneously shown, and the z-direction displacement is more obvious and mainly concentrated at the free end of the template. The displacement values and areas of Streamflow-O and Streamflow-T were relatively low and limited, but the displacement in Rayware-T was more severe, even occurring in the x- and y-directions. **b** A bar chart based on the distributions of test points and their groups was drawn, and the results were consistent with those in (**a**). Moreover, the different resins did not significantly influence the dimensional accuracy. *p < 0.05, **p < 0.01, ***p < 0.001

The means and standard deviations of the displacements for all 22 test points of each sample are shown in Table 1. All the data were displayed separately in four aspects, including the x-, y-, and z-directional displacements and the dxyz displacement. In this study, Streamflow had the best dimensional printing accuracy (Streamflow-O: 32.6 ± 59.3 μm; Streamflow-T: 31.3 ± 60.2 μm), the accuracies of Shapeware-T (56.4 ± 106.3 μm) and Polydevs-T (55.3 ± 114.2 μm) were moderate, and Rayware-T presented the worst dimensional accuracy of the surgical templates immediately after production (96.4 ± 191.4 μm). The actual and nominal coordinates of all 22 test points are shown in Fig. 1a, and the results revealed that all the templates exhibited positive displacements in the z direction (i.e., the vertical direction). The obvious displacements were mainly concentrated on the free end (obvious displacement > mean + SD, marked with yellow circles), and the test points near the implant sleeve exhibited slight displacements (mean < slight displacement < mean + SD, marked with green circles). However, the z displacements of Streamflow-O and Streamflow-T occurred mainly on the right side of the template, whereas those of Shapeware-T, Rayware-T and Polydevs-T occurred on both sides of the template.

**Table 1:** Displacements of all templates at different standing times

	Streamflow-O								Streamflow-T							
	X (μm)		Y (μm)		Z (μm)		XYZ (μm)		X (μm)		Y (μm)		Z (μm)		XYZ (μm)	
	Mean	SD	Mean	SD	Mean	SD	Mean	SD	Mean	SD	Mean	SD	Mean	SD	Mean	SD
0 w	5.9	3.5	7.3	3.7	80.8	85.0	32.6	59.3	6.6	5.0	6.5	3.9	84.5	80.7	31.3	60.2
1 w	5.0	3.0	4.9	3.1	79.4	80.9	29.8	58.4	5.0	4.3	4.5	3.1	76.5	65.5	28.7	50.8
2 w	6.2	3.3	6.9	3.9	78.1	81.7	30.4	57.9	6.1	6.0	8.0	4.2	67.4	63.7	27.2	46.6

	Shapeware-T								Rayware-T								Polydevs-T							
	X (μm)		Y (μm)		Z (μm)		XYZ (μm)		X (μm)		Y (μm)		Z (μm)		XYZ (μm)		X (μm)		Y (μm)		Z (μm)		XYZ (μm)	
	Mean	SD	Mean	SD	Mean	SD	Mean	SD	Mean	SD	Mean	SD	Mean	SD	Mean	SD	Mean	SD	Mean	SD	Mean	SD	Mean	SD
0 w	5.9	4.9	8.8	4.9	154.6	139.8	56.4	106.3	8.4	5.4	8.8	5.9	271.9	253.0	96.4	191.4	4.8	2.7	8.0	3.5	153.1	158.0	55.3	114.2
1 w	5.1	4.4	5.5	4.3	158.5	143.8	56.3	109.9	7.0	5.5	8.5	5.8	268.4	249.4	94.6	189.0	4.9	3.1	5.3	2.5	157.2	149.3	55.8	111.9
2 w	6.8	5.7	8.8	5.7	188.0	152.0	67.9	122.0	8.3	5.6	8.3	6.0	262.3	244.9	93.0	185.0	5.8	3.3	9.0	3.2	168.7	151.5	61.2	115.7

The average dxyz values of Streamflow-O, Streamflow-T, Shapeware-T, Rayware-T and Polydev-T are 32.6 μm, 31.3 μm, 56.4 μm, 96.4 μm, and 55.3 μm, respectively. Moreover, the average dxyz appeared to be negligible after 2 weeks of storage, and the displacements in the z direction were relatively distinguishable and were only approximately 30 μm at week 1 and 10 μm at week 2

The displacement distributions of all 22 test points are shown in Fig. 1b. The bar chart reveals that the dimensional accuracy difference among all groups was reflected mainly in the z-axis, in which the Streamflow-O and Streamflow-T values were significantly lower than those of the other groups (*p* < 0.001). Additionally, the displacements of the test points near the sleeve were lower than those of the free-end region, which was consistent with the above-stated results. In this study, no significant difference was found between the dimensional accuracies of the different resins (*p* > 0.05).

### Dynamic deformation

**Fig. 2:**
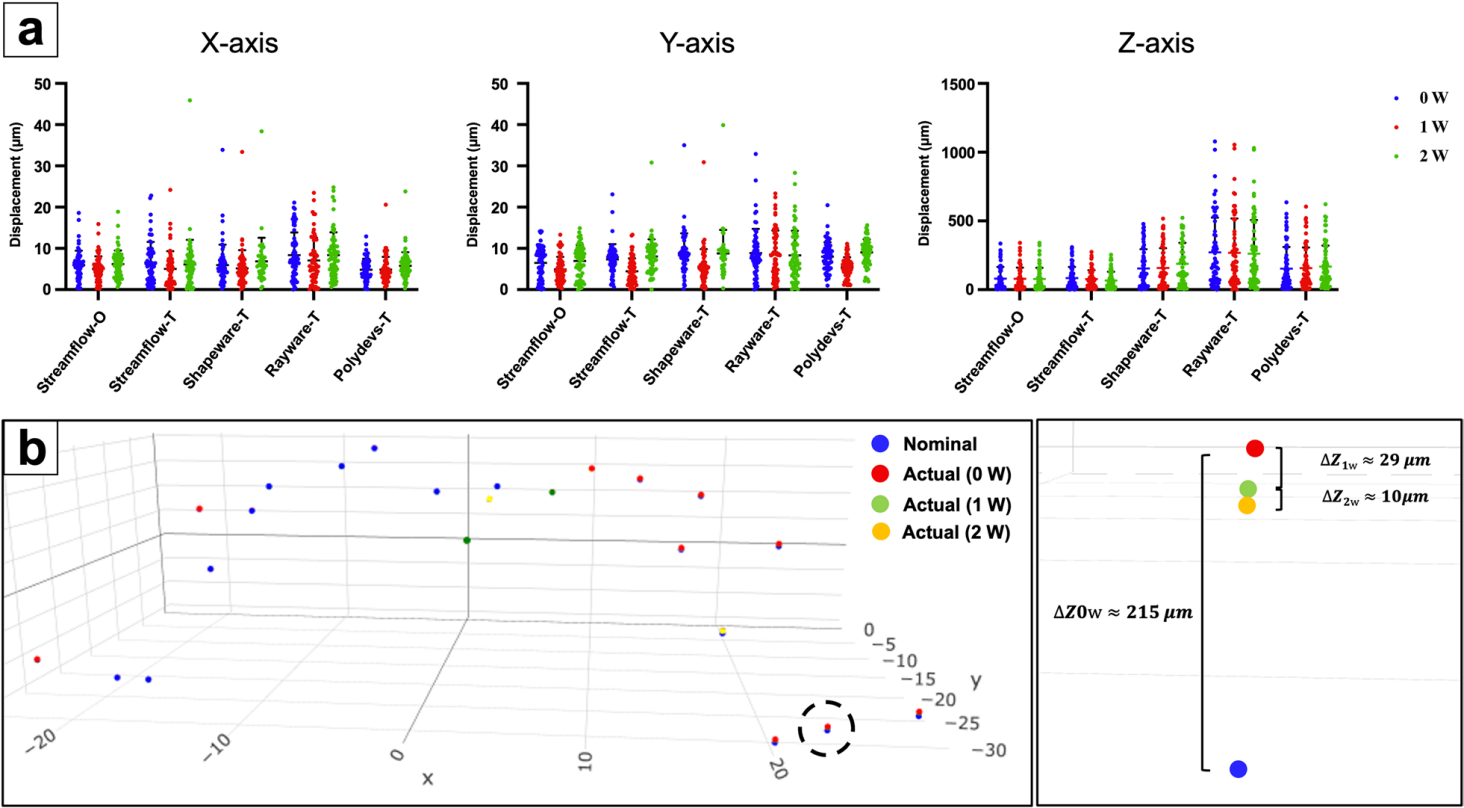
Deformation of all templates along the x-, y-, and z-axes after different standing times. **a** No significant deformation, which was mainly determined by the printing dimensional accuracy (0 w), occurred after 2 weeks of storage. **b** Representative results revealed that the only discernible displacement was exhibited in the z-direction; this deformation measured approximately 30 μm after one week and 10 μm after the second week

To evaluate the possible dynamic deformation, all test points in the different groups were successfully observed at three standing time points (0 w, 1 w, and 2 w) by the CMM. As shown in Table 1, the mean dxyz displacements were 31.3–96.4 μm at week 0 (equivalent to dimensional accuracy), 28.7–94.6 μm at week 1 and 27.2–93 μm at week 2, and no significant dynamic deformation was observed for any of the samples (*p* > 0.05, Fig. 2a). Considering that the performances of all the groups were basically consistent, the results of Streamflow-T were regarded as representative of the results (Fig. 2b). In particular, the largest deformation was observed at the postfabrication timepoint (0 w), which was mainly determined by the dimensional accuracy of the printing process (ΔZ_0w_ ≈ 215 μm, inset of Fig. 2b). The z displacements in the free-end region after 1 week and 2 weeks of storage were almost 29 μm (ΔZ_1w_) and 10 μm (ΔZ_2w_), respectively (inset of Fig. 2b). The deformations in the x- and y-axes were both minimal and did not significantly differ during the 2-week storage period (*p* > 0.05).

## Discussion

Manufacturing methods and providers of DLP-printed surgical templates have increased in popularity in recent years; however, the accuracy of these different production procedures remains to be determined [12]. To our knowledge, this is the first study using a CMM to conduct a high-precision dimensional accuracy analysis and an in situ observation of dynamic deformation for 3D-printed surgical templates.

**Scheme 1:**
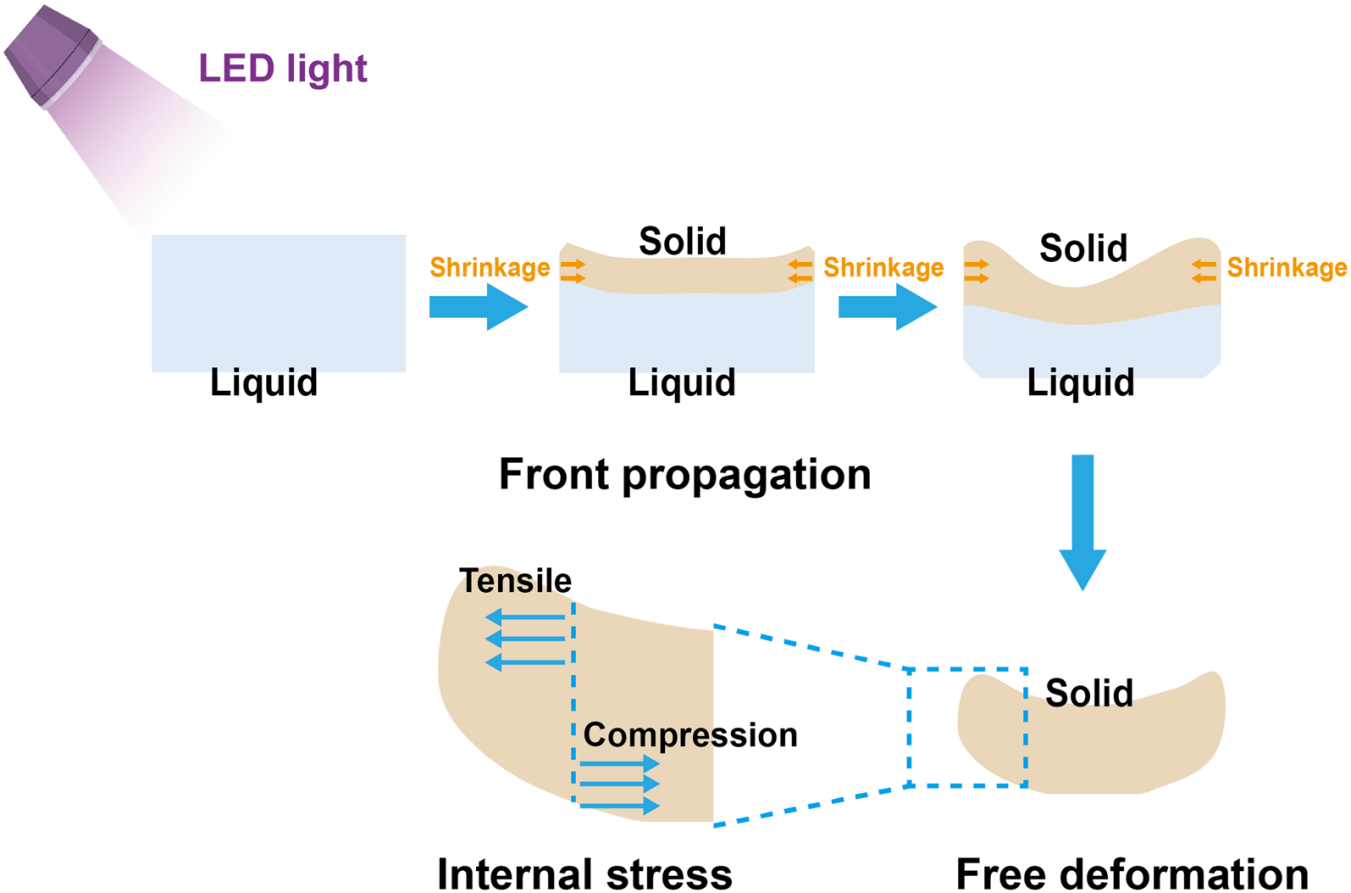
Schematic diagram of volume shrinkage-induced bending. The mechanism is as follows: sequential shrinkage occurs during the frontal photopolymerization of a polymer sheet, and the internal stress developed during this process drives the sheet to bend

In this study, the mean dimensional accuracy (dxyz) for all templates was within 100 μm (31.3–96.4 μm), which is basically consistent with or even better than the results reported in other studies of DLP-printed surgical templates (95 ± 36 μm [13]; 200 ± 110 μm [14]). This is also an acceptable range for clinical accuracy [15]. Further comparison revealed that the dimensional accuracies of Streamflow-O and Streamflow-T seemed to be better than those of the other groups, whereas the accuracy of Rayware-T was relatively poor. The dxyz was caused mainly by the positive z displacements on both sides, and especially the free ends, of the templates, whereas deviations in the horizontal direction (x- and y-axes) appeared to be negligible. Matta et al. reported the same conclusion, namely, that the highest deviations of all the produced templates based on the data of 13 patients were on the z-axis compared with the x- and y-axes (0.594 mm vs. 0.346 mm and 0.266 mm, respectively) [16]. Additionally, we specifically found that the dimensional deviations of the templates were caused mainly by the upward bending of the free-end region (positive z displacement). This is not surprising because numerous studies have reported this phenomenon and called it “volume shrinkage-induced bending”; the mechanism is that sequential shrinkage occurs during the frontal photopolymerization of a polymer sheet and the internal stress developed during the process drives the sheet to bend (Scheme 1), with this impact being more significant in the z plane [15, 17]. This also explains why the dimensional accuracy of the sleeve at the center of the model is much better. These results suggest that long-term surgical templates should be avoided on the premise of ensuring retention force. In addition, several studies have reported that altering the exposure energy [18] or build angle [19] can reduce shrinkage-induced bending, but further studies are still needed to determine the specific processing parameters.

There was no significant difference between the dimensional accuracies of opaque (Streamflow-O) and transparent resin-based templates (Streamflow-T), even if they were produced by the same 3D printer. However, the opaque resin used in this study was reported to have a greater bending strength (83.2–88.2 MPa) and notched impact strength (32.0–36.6 J/m) than the transparent resin (75.4–83.6 MPa, 20.3–23.7 J/m, according to information provided by the manufacturer), which may increase the strength of the opaque template when it is applied intraorally. The drawback is that the opaque template obstructs the observation of the surgical field. The above conclusions need to be treated with caution considering that mechanical strength measurements were not conducted in this study.

In this study, the CMM successfully performed long-term and in situ analysis for dynamic deformation observation. No significant deformation occurred among all DLP-printed templates after 2 weeks of storage, and the absolute values of the z-direction deviations were only approximately 30 μm at week 1 and 10 μm at week 2. This finding suggests that the discrepancy between DLP-printed templates is determined mainly by their immediate processing accuracy and that their dimensional stability is promising even without the use of a sponge or other support materials. Since there is currently no CMM-based dimensional accuracy analysis comparing this method with other 3D printing technologies, such as stereolithography appearance (SLA) and laser cladding deposition (LCD), it is still too early to draw the conclusion that DLP-printed templates are more attractive options, but we cautiously consider the accuracy and stability of the dimensions exhibited by DLP templates in this study to be clinically acceptable. Subsequent systematic in vitro and in vitro analyses addressing this issue are needed in the future.

The aim of this study was to demonstrate the viability of a CMM for the geometric analysis of 3D-printed surgical templates. However, several limitations must be acknowledged. First, the accuracy results for templates produced by different printers need to be considered with caution because of the difficulty in standardizing the printer parameters and resins set by their own manufacturers. Second, the addition of landmarks and a base to the templates, which facilitated CMM detection, may also have potentially impacted the results of this study, although we tried to minimize their volumes. Third, the CMM can conduct high-precision and in situ detection but can measure only the selected test points. Thus, it is impossible to collect global surface information for analysis, such as via an optical scanner, which means that the accuracies of other unselected points remain unknown. Last, the criteria for defining slight versus obvious displacement are based on whether it is greater than the mean + 1 standard deviation. There is currently no uniform standard for displacement evaluation.

## Conclusion

Owing to the limitations of this study, the Euclidean displacement (dxyz, representation of dimensional accuracy) ranged from 31.3 to 96.4 μm for all DLP-printed templates, and this impact was more significant in the z plane. The different resins and the 2-week standing time did not significantly influence the dimensional accuracy or stability. Through this study, a CMM is preliminarily demonstrated as a promising method for high-precision and in situ analysis of the dimensional accuracy and dynamic deformation of 3D-printed implant surgical templates.

## Methods

### Data acquisition

This in vitro study was conducted with the case of a clinical patient exhibiting a single edentulous space at the left maxillary central incisor region (21, FDI dental numbering system) who was scheduled for implant placement. This study was approved by the Ethics Committee of Xi'an Jiaotong University Stomatology Hospital, Shaanxi, China (2024-XJKQIEC-QT-0002-001). Preoperative CBCT data were obtained via the following machine parameters: tube voltage, 100 kV; tube current and rotation period, 100 mAs; field of view, 16 cm × 10 cm; and slice width, 300 μm; KaVo Company, Germany). The optical impression was measured across the dental arch using TRIOS® 3 (3 Shape, Copenhagen, Denmark) without any powder. Prior to scanning, the scanner tip was calibrated and preheated as instructed by the manufacturer.

### Surgical template design

**Fig. 3:**
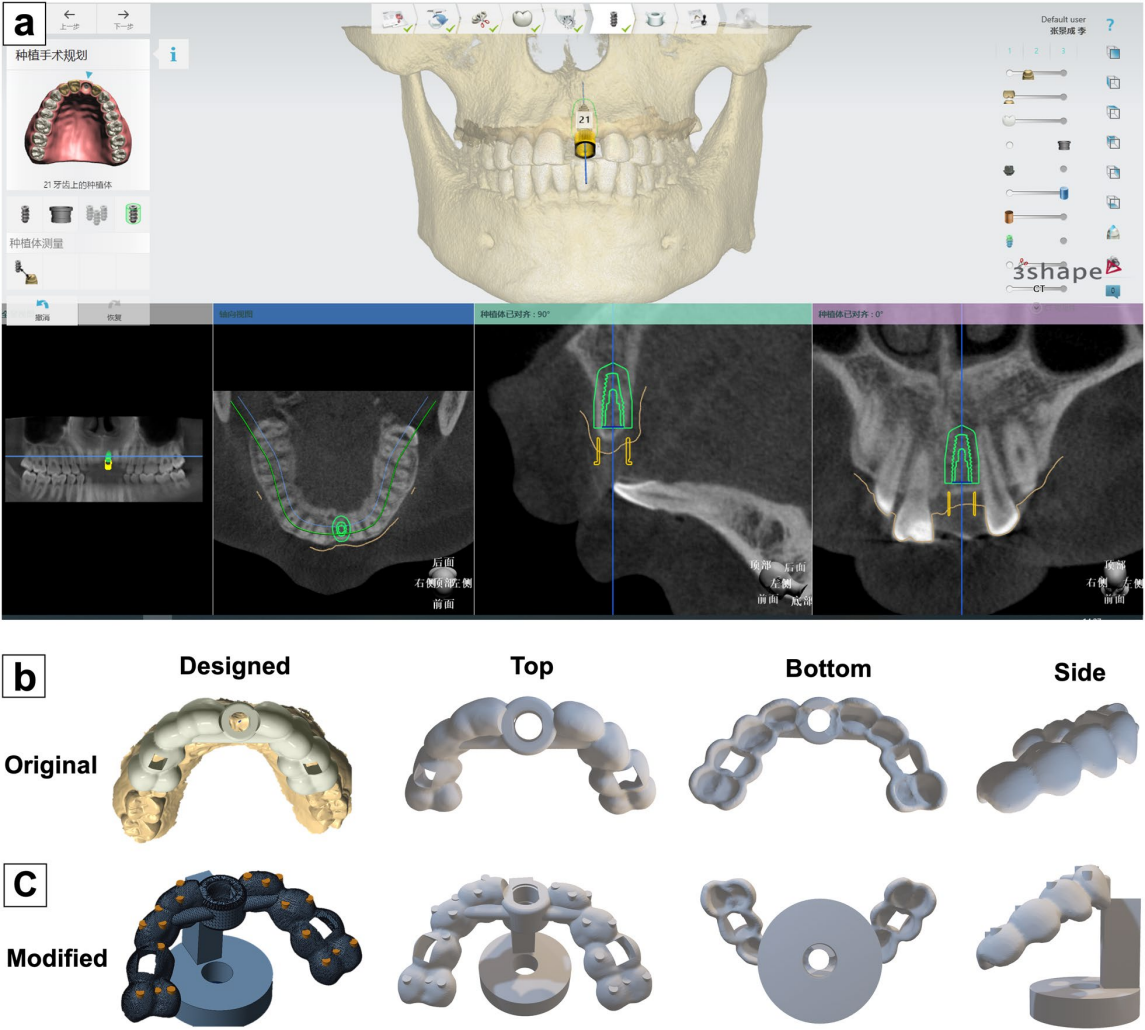
Design and modification of the template model in STL format. **a** Template design interface of the 3Shape implant studio software, in which the Straumann BLT (4.1 mm × 10 mm) implant was virtually inserted in region 21; **b**, **c** original and modified STL model files were displayed from three perspectives, in which 18 cylindrical landmarks and a round base were added

For the digital planning of the implant position, the standard tessellation language (STL) file of the optical scan and digital imaging and communications in medicine (DICOM) data of CBCT were uploaded into a dedicated software program (Implant Studio; 3Shape). Both data files were digitally matched by using easily defined anatomic landmarks, primarily the natural teeth and attached gingiva. The 3D implant position was planned with an appropriate length and diameter (Fig. 3a, bone level tapered implant; 10 mm length, diameter: 4.1 mm; Institut Straumann AG). For the digital design, the surgical template was fixed on both sides to 4 adjacent teeth. Observation windows were set at the cusps of the right maxillary canine and the left maxillary first premolar (Fig. 3b).

### Addition of the measurement landmarks and base

The STL format has become standard for data input of all types of rapid prototyping systems, but polygonal areas of the STL model with millions of triangles often require long processing times, and it is difficult for CMM to determine the nominal coordinates [20]. To facilitate measurement, we postprocessed the STL model by randomly creating 18 landmarks on both sides of the template surface (the dentition part of the template) via Creo Parametric 4.0 software (PTC; Needham, MA, USA). Each landmark was a cylinder with a diameter of 2 mm, and the upper plane was fully exposed. By analyzing 6 points on the upper edge of the cylinder with the CMM probe, the coordinates of the center point (as the test point) were determined, and CMM calibration was also performed simultaneously by calculating the actual diameter of the cylinder (1 μm tolerance was allowed, 1.999–2.001 mm). In addition, we randomly selected 4 test points at the surface of the sleeve without adding any landmarks because the topology of this area was flat and easy to fit into a plane. Thus, a total of 22 test points were set on each template. Moreover, a column with a circular base was also created on the STL model to stabilize the template on the platform when performing the measurements (Fig. 3c).

### Manufacturing of surgical templates

**Fig. 4:**
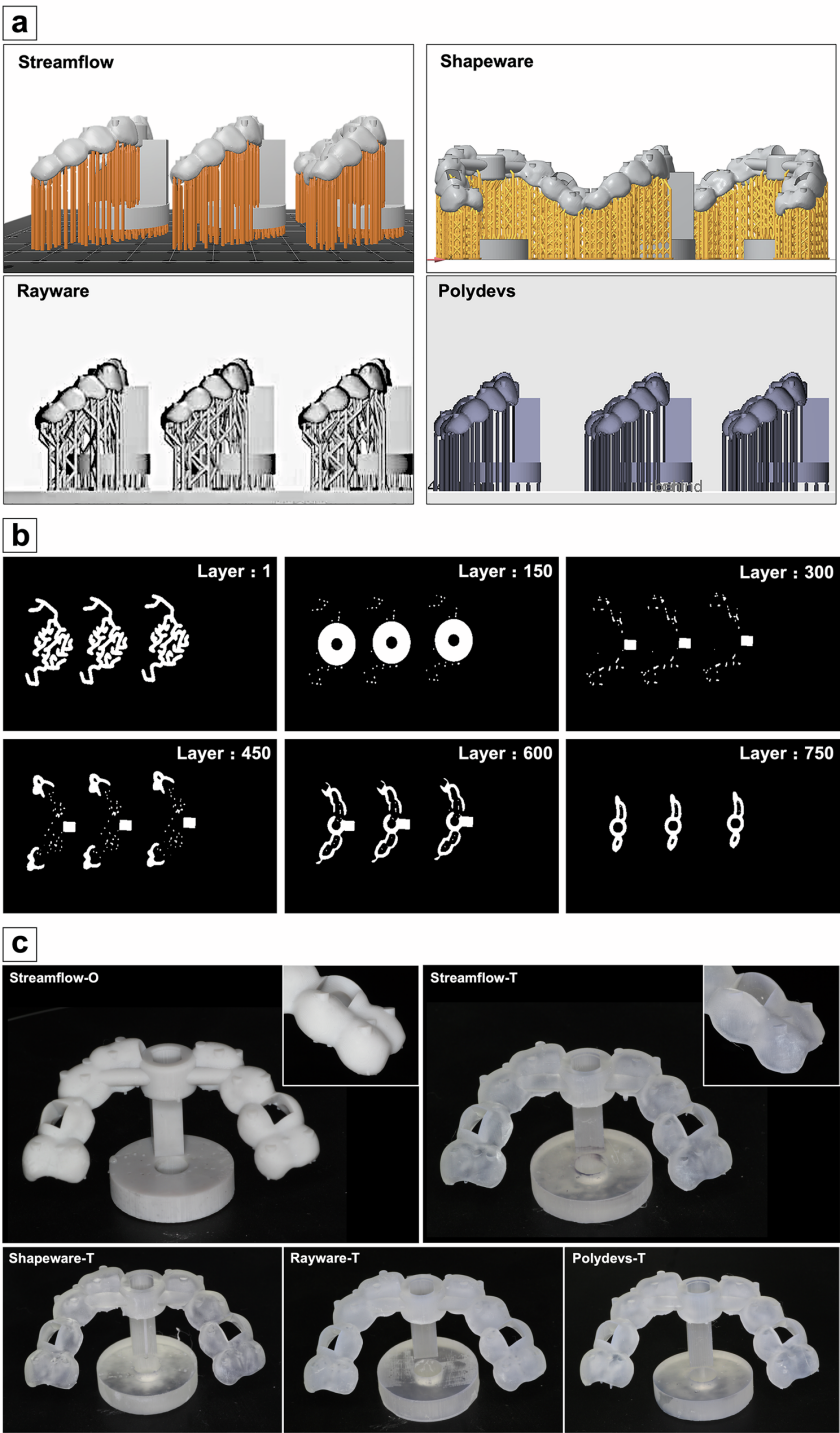
Preprocessing settings in the slicing software and all the fabricated templates. **a** The STL model was nested on the build platform at 0° followed by support structure addition in the slicing software; **b** the model was sliced at a layer thickness of 50 μm, and the interfaces of the different layers are shown; **c **the modified templates were successfully printed, including the landmarks and round base

**Scheme 2:**
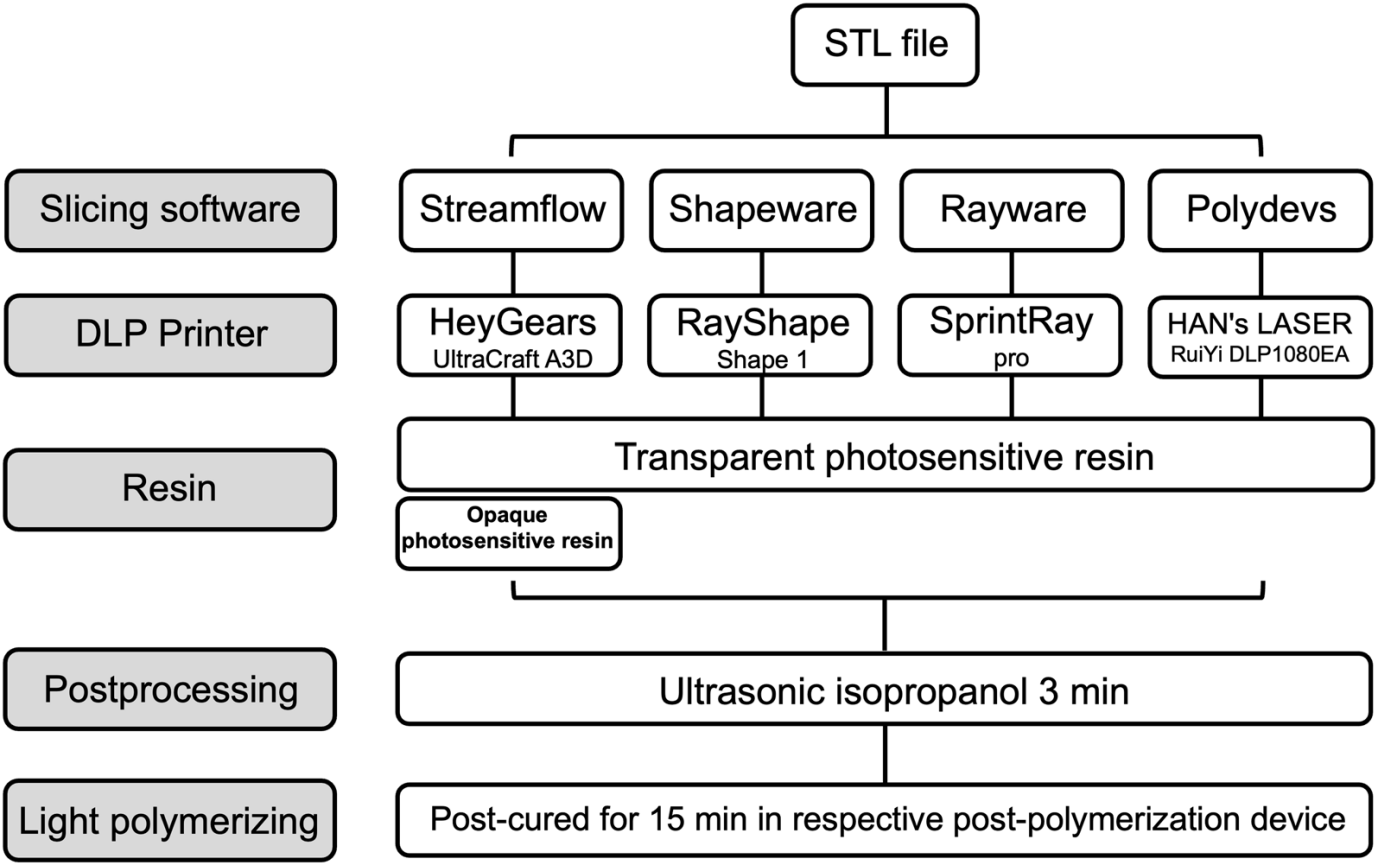
Manufacturing workflow of the template. The 3D printing digital process consists of slicing, printing, postprocessing and light polymerizing

The postprocessed STL file was imported into four different 3D slicing software programs (1. Streamflow, HeyGears V2.4.25, Guangzhou, China; 2. Shapeware, RayShape, Suzhou, China; 3. Rayware, SprintRay, USA; 4. Polydevs, Uniontech, Shanghai, China) and nested on the build platform at 0°, followed by support structure addition (Fig. 4a). Then, the samples were sliced at a layer thickness of 50 μm (Fig. 4b). G-codes were generated and transmitted to their own digital light processing (DLP) printers (1. HeyGears, UltraCraft A3D, Guangzhou, China; 2. RayShape, Shape 1, Suzhou, China; 3. SprintRay, pro, USA; 4. HAN's LASER, RuiYi DLP1080EA, Shenzhen, China). Two types of medically certified denture resins (opaque and transparent) were used on the basis of the manufacturer’s safety data sheet (Opaque resin: HeyGears printer: Model HP UV 2.0, HeyGears, China; Transparent resins: 1. HeyGears printer: Surgical Guide UV, HeyGears, China; 2. RayShape printer: SG resin, RayShape, China; 3. SprintRay printer: Surgical Guide 3, SprintRay, USA; 4. Han laser printer: Leyi D031, Hangzhou Leyi New Material Technology Co., Ltd., China). Next, all the samples were ultrasonically twice cleaned in 99% isopropanol for 3 min and then postcured for 15 min in a light chamber with an ultraviolet light emitting diode that ranged from 360 to 440 nm and peaked at approximately 385 nm. Finally, the support structures were removed, and the fabricated templates were divided into five groups according to the applied slicing software and resin (opaque or transparent), named “Streamflow-O”, “Streamflow-T”, “Shapeware-T”, “Rayware-T” and “Polydevs-T” (N = 3, Fig. 4c). The above workflow is shown in Scheme 2.

### CMM measurement

**Fig. 5:**
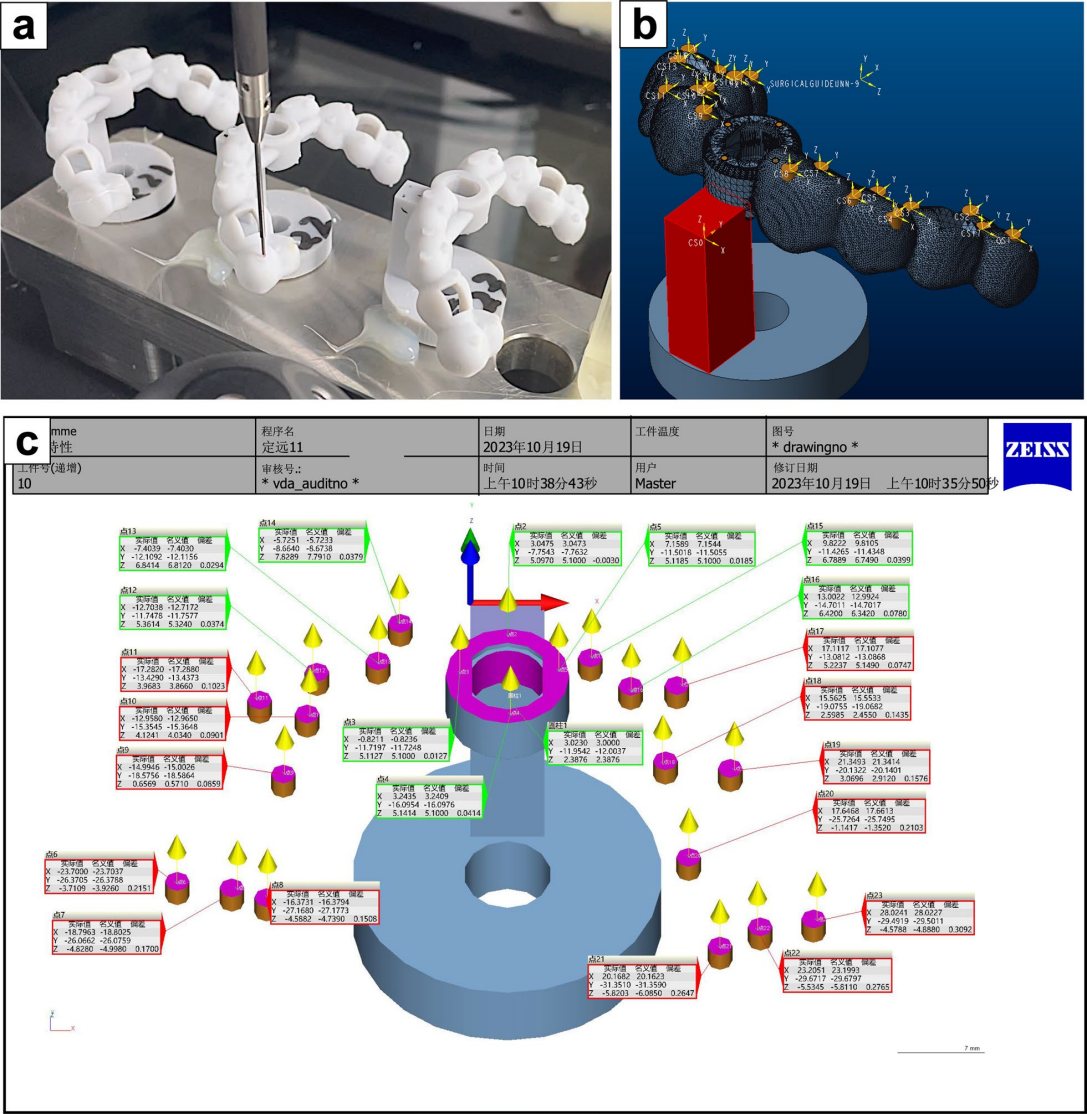
Measurement and output results of the CMM. **a** Each group consisted of three identical samples, which were fixed on the same test platform. The CMM probe measured the coordinates of 22 test points according to the preset program. **b** All 22 test points are highlighted in yellow and are composed of 18 cylindrical landmarks randomly distributed on both sides of the template and 4 points on the sleeve. The origin was set at the top corner of the base connector, and a rectangular coordinate system was established. **c** The CMM automatically calculated the actual and nominal coordinates and displacement values after the measurement was completed

All the samples were fixed on a CMM platform (O-INSPECT 543, Zeiss, Germany) by using epoxy resin (Zhejiang Zhongli Petrochemical Co., Ltd., China). Figure 5a), and the test process was performed as previously reported [21]. (1) Coordinate system establishment: the model was imported into the CMM software (Calypso 5.4.20, Zeiss, Germany), followed by selection of the origin point and establishment of the measurement coordinate system (point CS0 shown in Fig. 5b in red); (2) Determination of the test points: a total of 22 test points were set in software, including 18 cylindrical landmarks and 4 points on the implant sleeve (the yellow points in Fig. 5b); (3) Element construction: the software programs calculated the position and dimensional characteristics of elements on the basis of the test-point coordinates; (4) Data computation: the software program obtained each element’s geometric dimensions and positional tolerances and calculated the nominal coordinates of test points referring to the established coordinate system; (5) Computer numerical control (CNC) operation: after configuration of the CMM parameters, such as the safety planes, retraction distances, probe types, and operating speeds, the automatic CNC operation of the CMM for model measurement was initiated (an additional movie file shows this step in more detail, Additional file 1); (6) Results output: the software calculated the displacements between the nominal (designed template) and actual coordinates (printed template) of all test points on the x-, y-, and z-axes (Fig. 5c), and the Euclidean distance (dxyz) was regarded as the representation of global dimension accuracy:

$$\text{dxyz }=\sqrt{{({x}_{actual}-{x}_{nominal})}^{2}{+({y}_{actual}-{y}_{nominal})}^{2}+{({z}_{actual}-{z}_{nominal})}^{2}}$$

In this study, the above process was repeated three times for each sample, and three time points were set to observe the possible dynamic deformation of the surgical template immediately post-production and after 1 week and 2 weeks of storage (0 w, 1 w, and 2 w).

### Statistical analysis

All the statistical analyses were performed in SPSS Statistics version 26.0 (SPSS, Chicago, IL, USA). The values are presented herein as the means ± standard deviations. One-way ANOVA was used for comparisons of the data among multiple groups, whereas the least significant difference (LSD) test was used for further comparisons between two groups. All tests for significance were two-sided, and *p* < 0.05 was considered statistically significant. All the bar charts were generated by GraphPad PRISM software version 9.0 (GraphPad Software, Inc., San Diego, US). All 3D coordinate diagrams consisting of the actual and nominal coordinates of all test points were created by Rstudio (RStudio, Boston, MA, USA).

## Supplementary Information

**Additional file 1.**

## Data Availability

Available from the corresponding author on reasonable request.

## Abbreviations

3D printing: Three-dimensional printing
ANOVA: Analysis of variance
CBCT: Cone beam computed tomography
CMM: Coordinate measuring machine
STL: Stereolithography
DLP: Digital light processing
FDI: Fédération Dentaire Internationale
SLA: Stereolithography appearance
LCD: Laser cladding deposition
Streamflow-O: Streamflow software with opaque resin
Streamflow-T: Streamflow software with transparent resin
Shapeware-T: Shapeware software with transparent resin
Rayware-T: Rayeware software with transparent resin
Polydevs-T: Polydevs software with transparent resin
